# Ozone Pollution in the Western Yangtze River Delta During the 2020 and 2021 Warm Seasons: Roles of Meteorology and Air Mass Transport

**DOI:** 10.3390/toxics13080670

**Published:** 2025-08-09

**Authors:** Yuchen Wang, Ming Wang, Feng Ding, Xueqi Chen, Liangyu Zhang

**Affiliations:** 1 Collaborative Innovation Center of Atmospheric Environment and Equipment Technology, Jiangsu Key Laboratory of Atmospheric Environment Monitoring and Pollution Control, School of Environmental Science and Engineering, Nanjing University of Information Science & Technology, Nanjing 210044, China; 202283300474@nuist.edu.cn (Y.W.); 202312480084@nuist.edu.cn (X.C.); 2 Jiangsu Nanjing Environmental Monitoring Center, Nanjing 210041, China; 18951651491@163.com (F.D.); zlyuuuu@163.com (L.Z.)

**Keywords:** surface ozone, non-methane hydrocarbons, air mass transport, potential source regions, ozone formation sensitivity

## Abstract

Surface ozone (O_3_), a key hurdle in air quality improvement in China, often displays regional pollution characteristics. This study investigated the influence of meteorological conditions and air mass transport on O_3_ and non-methane hydrocarbons (NMHCs) concentrations in Nanjing, located in the western Yangtze River Delta (YRD) region of China during April–September of 2020 and 2021 based on online observations of O_3_ and its precursors and meteorological conditions, backward-trajectory analysis, and an observation-based box model (OBM). O_3_ concentrations rose with temperature, albeit non-linearly. Southeastern trajectories constituted the most dominant air mass transport pathway (29.3%) and were associated with the highest O_3_ concentrations. The concentration-weighted trajectory analyses of O_3_ and NMHCs during four O_3_ pollution episodes suggested that urban/industrial areas in central and eastern YRD were potential source regions. The OBM results indicated that while O_3_ sensitivity was dominated by the NMHCs-limited regime, the relative contributions of three O_3_ sensitivity regimes varied across air mass trajectory clusters. The southeastern air masses with long-range transport showed the highest frequency of the transition and NO_x_-limited O_3_ sensitivity regimes. These findings underscore the crucial role of regional air mass transport not only in determining O_3_ and NMHCs concentrations but also in shaping O_3_ formation sensitivity, highlighting the necessity of implementing regionally coordinated control strategies for effective O_3_ and NMHCs pollution mitigation.

## 1. Introduction

Over the past decade, primary air pollution issues such as sulfur dioxide (SO_2_) have improved significantly in China; however, secondary pollutants such as surface ozone (O_3_) still constitute a pressing concern [1,2,3,4]. Numerous studies have indicated that elevated concentrations of O_3_ can harm human health [5,6], reduce crop yields [7], and cause other detrimental effects. As a secondary pollutant, O_3_ formation is influenced by precursor emissions and meteorological conditions [8,9] that pose a considerable challenge to control efforts [10]. In recent years, O_3_ pollution has become one of the main obstacles in improving air quality in China [11].

Studies have shown that surface O_3_ often displays typical regional pollution characteristics [12], suggesting that atmospheric transport significantly influences the spatiotemporal distribution of O_3_ concentrations [13]. Firstly, O_3_ is primarily formed through photochemical reactions involving volatile organic compounds (VOCs) and nitrogen oxides (NO_x_), and its formation is thus strongly influenced by emission intensity and spatiotemporal distribution of VOCs and NO_x_ [14,15]. Furthermore, air mass transport processes can also influence photochemical reaction intensity and prevailing meteorological conditions [16,17,18]. Several studies have documented the significant impact of air mass transport on O_3_ concentrations. For example, Sahu et al. (2021) demonstrated the influence of long-range atmospheric transport from outside China on O_3_ levels in its eastern coastal and southwestern regions [19]. Gong et al. (2020) reported that in the Beijing–Tianjin–Hebei region, air mass transport contributed to a 36% increase in O_3_ concentrations [20]. Li et al. (2021) observed in the Yangtze River Delta (YRD) that high concentrations of O_3_ originating from within Shanghai spread horizontally and were subsequently transported downward from the free troposphere to northern YRD, significantly increasing regional O_3_ concentrations [21]. Similarly, Li et al. (2024) found that during autumn 2019, 42% of O_3_ concentrations in the Pearl River Delta (PRD) region was attributed to air mass transport mostly from Guangdong province and eastern China [22]. Besides the direct influence on O_3_ concentration, air mass transport can also indirectly affect regional O_3_ levels by altering the extent of photochemical reactions [23]. Some studies indicated that shifts in the ratio of VOCs versus NO_x_ during air mass transport can modify the sensitivity of O_3_ formation to VOCs and NO_x_ [24,25,26], while others found the chemical composition of VOCs also changed during air mass transport, further impacting O_3_ formation [27,28].

The YRD region, a major urban agglomeration in eastern China, is one of the areas with the highest O_3_ concentrations in the country [29]. Within the YRD, O_3_ concentrations typically elevated in the central and western areas [18,30]. Since 2016, southern cities in Jiangsu Province have experienced notable increases in O_3_ concentrations, with some periods showing higher levels than other major YRD cities such as Shanghai and Hangzhou [30]. Nanjing, a central city located in western YRD and southern Jiangsu, has been particularly affected [31]. From 2017 to 2019, the number of O_3_ exceedance days in urban Nanjing remained above 60 annually, with exceedance rates over 15% (https://sthjj.nanjing.gov.cn/index.html (accessed on 21 March 2025)). While O_3_ pollution saw a slight dip in 2020 (6.9% year-on-year decrease in exceedance rate), it unfortunately rebounded in 2021 and 2022. Despite some observed reductions in concentrations in 2023 and 2024, O_3_ remains a significant contributor to air pollution in Nanjing (https://sthjj.nanjing.gov.cn/index.html (accessed on 21 March 2025)).

To assess the impact of meteorological conditions and air mass transport on urban O_3_ in Nanjing, this study analyzed the diurnal variations of O_3_ and its precursors concentrations, their relationship of temperature and wind, and investigated major pathways of air mass transport based on air mass backward-trajectory analysis. Four O_3_ pollution episodes occurring between April to September in 2020 and 2021 were then selected to further analyze the impact of synoptic conditions on O_3_ and investigate the sensitivity of O_3_ with NO_x_ and non-methane hydrocarbons (NMHCs) using an observation-based box model (OBM). Potential source regions of O_3_ and NMHCs in these four episodes were identified using the concentration-weighted trajectory (CWT) method. Furthermore, concentrations of O_3_ and its precursor, along with the O_3_-NMHCs-NO_x_ sensitivity was compared across various air mass back-trajectory clusters to discuss the influence of air mass transport. The results of this study can provide a scientific basis for O_3_ pollution prevention in Nanjing and YRD, while enhancing our understanding of how air mass transport influences O_3_ formation.

## 2. Materials and Methods

### 2.1. Field Observations

**Figure 1:**
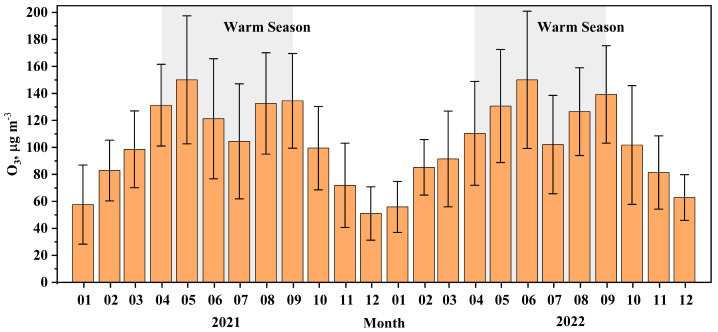
The monthly average ozone concentration in Nanjing during 2020 and 2021.

This study focuses on the high O_3_ pollution season (April–September) (Figure 1) during 2020 and 2021, following the implementation of the Three-Year Action Plan for the Battle of the Blue Sky (2018–2020). Hourly concentrations of O_3_, NMHCs, NO_x_, carbon monoxide (CO), and meteorological parameters were observed at an urban site (NJ, 118.76°E, 32.06°N) in Nanjing, Jiangsu Province, a central city within the YRD region of China. This site is located approximately 300 km west of Shanghai, 250 km northwest of Hangzhou in Zhejiang Province, and 170 km east of Hefei in Anhui Province. The surroundings of this site are residential and commercial buildings, with no significant local industrial emission sources in the immediate vicinity. The nearest road network includes the West Inner Ring Road (approximately 250 m to the east), the North Inner Ring Road (approximately 1.5 km to the north), and an expressway (approximately 1.3 km to the west). The two major petrochemical industrial zones in Nanjing are located approximately 20 km northeast of the site. Detailed descriptions of this site and measurement methods for O_3_ and its precursors were provided in Lu et al. (2023) [32]. In brief, ambient NMHCs were pre-concentrated using a dual-channel ultra-low-temperature cold trap, thermally desorbed, and then analyzed using a gas chromatography system with a flame ionization detector and mass spectrometry (GC–MS/FID) (Agilent Technologies, Inc., Santa Clara, CA, USA). A total of 57 NMHCs, comprising 28 alkanes, 11 alkenes, acetylene, and 17 aromatics, were measured during the 2020 and 2021 campaigns (Appendix A). Details about this system following the protocol of Wang et al. (2014) [33]. O_3_, NO/NO_2_, and CO were measured with Model 49i ultraviolet absorption, Model 42i chemiluminescence, and Model 48i infrared analyzers (Thermo Fisher Scientific, Waltham, MA, USA), respectively.

### 2.2. Backward-Trajectory and Concentration-Weighted Trajectory (CWT) Analysis

Air-mass backward-trajectories and CWT analyses were performed using MeteoInfo (Version 3.9.4) [34]. The inputted gridded meteorological data were obtained from the Global Data Assimilation System (GDAS) at a 0.1° × 0.1° resolution (ftp://arlftp.arlhq.noaa.gov/pub/archives/gdas1). In published literatures on O_3_ transport, air mass transport times for backward trajectories are typically set at 24 to 72 h (1–3 days) [21,35,36,37]. For this study, given our focus on air transport within the YRD region and eastern China, and considering the relative short lifetimes of NMHCs and NO_x_, we selected 12 h, 24 h, and 48 h trajectories for analysis. The time interval for each trajectory was set at one hour. The trajectory ending height was set at 500 m above ground level following the published literatures on air mass transport [36,37] and the reported boundary layer height in Nanjing [38]. The trajectories for each air mass transport time were then clustered into six types based on Euclidean distance.

In this study, the weighted concentration-weighted trajectory (WCWT) method was applied to identify potential source regions of O_3_ and NMHCs during four O_3_ pollution episodes (detailed dates are provided in Section 3.3.1). The WCWT value for each grid cell (ij) is calculated using the following equations:

(1) $${WCWT}_{ij}=W_{ij}\times {CWT}_{ij}$$

(2) $${CWT}_{ij}=\sum_{l=1}^{M}\left(C_{l}\times \tau_{ijl}\right)/\sum_{i=1}^{n}\tau_{ijl}$$

(3) $$W_{ij}=\left\{\begin{array}{l} 1.00,\;n_{ij}>80\\ 0.70,\;{20<n}_{ij}\leq 80\\ 0.42,\;{10<n}_{ij}\leq 20\\ 0.05,\;n_{ij}\leq 10 \end{array}\right.$$

where M is the total number of trajectories; $C_{l}$ means the concentration of O_3_ or NMHCs observed upon arrival of trajectory l; $\tau_{ijl}$ represents the residence time of trajectory l in the ij grid cell; $n_{ij}$ is the number of trajectory endpoints in the ij grid cell. Higher WCWT values signify a greater potential for the respective grid cell to contribute to the observed levels of O_3_ or NMHCs at the NJ site. In accordance with China’s National Ambient Air Quality Standard, the O_3_ threshold concentrations for the four episodes were set at 160 μg m^−3^. This threshold corresponds to the Grade II standard for the daily maximum 8 h moving average (DMA-8h) O_3_ (general residential areas) and matches the Grade I standard for the daily maximum 1 h average O_3_ (special protection areas). The NMHCs thresholds were set as its average mixing ratios during each episode, specifically 13.2, 18.2, 11.0, and 13.2 ppbv.

### 2.3. Observation-Based Box Model (OBM)

To evaluate the formation sensitivity of O_3_ to its precursors, a 0-dimension observation-based box model (OBM) incorporating the carbon bond lumped chemical reaction mechanism (CB05) was employed to simulate the photochemical processes governing O_3_ formation and removal. The OBM was constrained by observed hourly averaged concentrations of NO, O_3_, CO, 57 NMHCs (lumped according to the CB05 mechanism), along with temperature, and relative humidity (RH) to simulate O_3_ formation and removal processes. Photolysis frequencies for O_3_, NO_2_, and other relevant species were determined using the Tropospheric Ultraviolet–Visible Radiation (TUV) model. Simulations were conducted for the period spanning 07:00 to 19:00. Further details regarding the OBM are available in Cardelino and Chameides (1995) [39] and Wang et al. (2020) [24]. In this study, an index of agreement (IOA) of 0.87 for observed versus simulated O_3_ concentrations suggested the good performance of OBM in O_3_ simulations.

The relative incremental reactivity (RIR) of individual precursors was calculated using the following equation:

(4) $$\mathrm{RIR}(X)=\frac{\left[P_{O_{3}-NO}\left(X\right)-P_{O_{3}-NO}\left(X-∆X\right)\right]/P_{O_{3}-NO}\left(X\right)}{∆S(X)/S(X)}$$

where $P_{O_{3}-NO}\left(X\right)$ represents the net O_3_ formation potential (i.e., the time integral of O_3_ formation rate during daytime 07:00–19:00) corresponding to the measured concentration of precursor X. $P_{O_{3}-NO}\left(X-∆X\right)$ means the net O_3_ formation potential resulting from a specific reduction (∆X) in the amount of X. S(X) denotes the source function of precursor X, primarily encompassing influences from emissions and horizontal air mass transport not accounted for within the OBM. ∆S(X) represents the change in S(X) resulting from ∆X. In this study, the ∆S(X)/S(X) was set as 10%.

The sensitivity of O_3_ formation to NMHCs and NO_x_ was classified into three regimes based on the relative incremental reactivity (RIR) values of anthropogenic NMHCs (AHC) and NO_x_ proposed by Xu et al. (2022) [40]. When RIR(NO_x_) is negative and RIR(AHC) is positive, it indicates that a reduction in NO_x_ leads to an increase in O_3_, while a reduction in anthropogenic NMHCs results in a decrease in O_3_. In this case, O_3_ formation is classified as NMHCs-limited, suggesting that reducing anthropogenic NMHCs is a more effective strategy for controlling O_3_ levels. When both RIR(NO_x_) and RIR(AHC) are positive, the sensitivity of O_3_ formation can be further classified based on the ratio of RIR(NO_x_) to RIR(AHC). Specifically, if the ratio RIR(NO_x_)/RIR(AHC) is less than 0.5, it indicates that O_3_ formation is still NMHCs-limited, meaning reductions in anthropogenic NMHCs are more effective than NO_x_ for O_3_ control. If the ratio exceeds 2, the O_3_ formation is considered NO_x_-limited, suggesting that reducing NO_x_ emissions would more effectively lower O_3_ levels. When the ratio falls between 0.5 and 2, the O_3_ formation is in transition regime, where both anthropogenic NMHCs and NO_x_ contribute significantly to O_3_ formation, and coordinated control of both precursors is necessary.

## 3. Results

### 3.1. Variation of O_3_ and Its Precursors and Influence of Meterological Conditions

#### 3.1.1. Diurnal Variations of O_3_ and Its Precursors Levels

For the period of April to September in 2020 and 2021, the average daily maximum 1 h (DMA-1h) O_3_ concentration was 149 ± 50.5 μg m^−3^. The number of days on which DMA-1h O_3_ concentrations surpassed the China’s National Ambient Air Quality Standard thresholds of 200 μg m^−3^ (Grade II) and 160 μg m^−3^ (Grade I) was 64 and 147, respectively. The average concentrations of NMHCs and NO_x_ were 16.7 ± 10.7 ppbv and 18.0 ± 14.5 ppbv, respectively. The average O_3_ concentrations during April–September of 2020 and 2021 were 86.0 ± 28.3 and 80.3 ± 28.7 μg m^−3^, respectively, with no statistically significant difference at the 0.05 level (two-tailed *t*-test) [32]. In contrast, NMHCs and NO_x_ levels significantly declined in 2021 compared to 2020, with relative decreases of 17.6% and 14.0%, respectively [32]. Temperature and wind speed between 2020 and 2021 also showed no significant differences at the 0.05 level (two-tailed t-test), with predominant wind directions from the east and southeast [32], indicating similar meteorological conditions between the two years.

**Figure 2:**
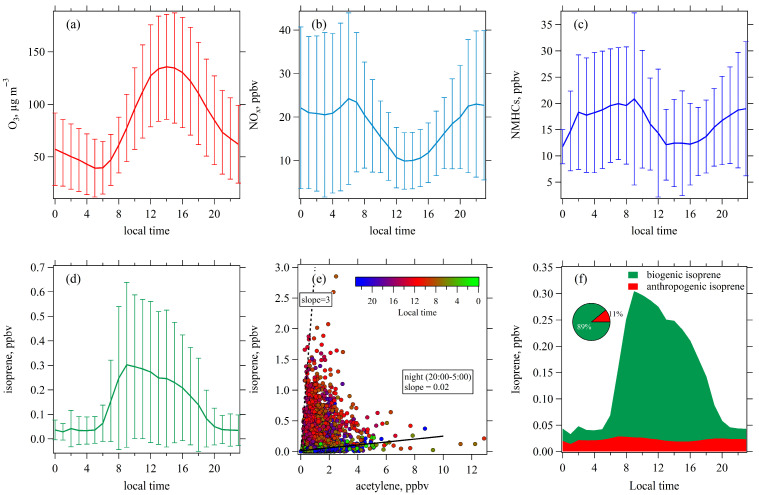
Average diurnal profiles of (**a**) O_3_, (**b**) NO_x_, (**c**) NMHCs, and (**d**) isoprene; (**e**) the relationship between isoprene and acetylene; and (**f**) the contributions of biogenic and anthropogenic emissions to isoprene during April–September in 2020 and 2021.

Figure 2 shows the average diurnal variation patterns of O_3,_ NO_x_, NMHCs, and isoprene levels. As depicted in Figure 2a, O_3_ concentrations peaked between 13:00 and 15:00. Conversely, NO_x_ and NMHCs concentrations exhibited peak values during the morning rush hour (05:00–07:00) and reached their lowest values in the early afternoon (13:00–15:00) (Figure 2b,c). Isoprene concentrations displayed a distinct diurnal profile compared to MMHCs, with high values observed from 10:00 to 16:00 and the lowest values occurring at night (Figure 2d). Furthermore, we analyzed the relationship between isoprene and acetylene (Figure 2e), which was mainly emitted from vehicular exhaust in urban Nanjing [24]. These two species showed poor correlations, with higher isoprene/acetylene ratios observed during daytime, with the highest isoprene/acetylene ratio approaching three. The ratio of anthropogenic isoprene to acetylene was estimated using two methods: (1) the slope of a linear regression between their concentrations during nighttime (20:00–23:00 and 00:00–05:00), and (2) the median of their ratios over the same period. These methods gave values of 0.024 and 0.020, respectively. Accordingly, we estimated anthropogenic isoprene as 0.02 times the measured acetylene, attributing the remaining isoprene to biogenic sources. Biogenic sources accounted for approximately 88% of total isoprene levels during daytime (Figure 2f), confirming that isoprene was predominantly biogenically emitted.

#### 3.1.2. Influence of Temperature on O_3_ and Its Precursors Levels

**Figure 3:**
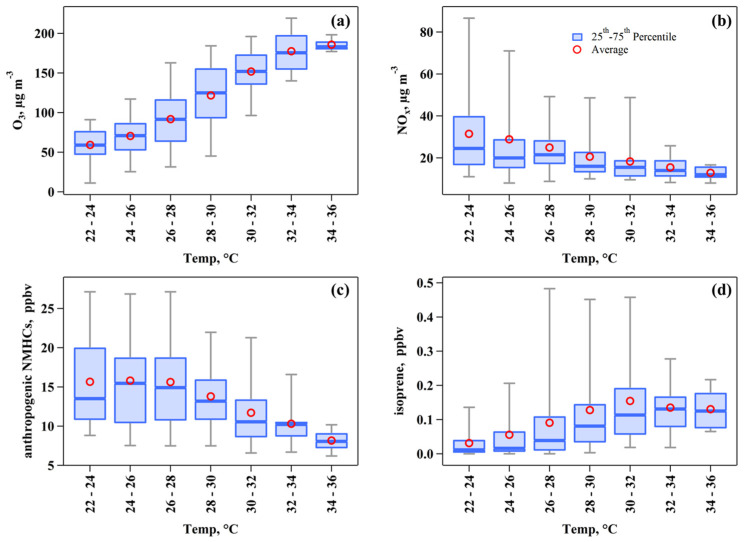
Temperature-dependent relationships of (**a**) O_3_, (**b**) NO_x_, (**c**) isoprene, and (**d**) anthropogenic non-methane hydrocarbons (NMHCs) observed from April to September in 2020 and 2021.

High temperatures frequently coincide with strong solar radiation, a combination that accelerates the photochemical reactions leading to O_3_ formation. Furthermore, these elevated temperatures enhance the release of NMHCs into the atmosphere, increasing evaporation from sources like paints, solvents, and gasoline, as well as promoting higher biogenic emission rates [41]. Figure 3a illustrates the relationship between O_3_ concentration and temperature observed in this study. O_3_ concentrations exhibited a positive, yet non-linear, relationship with temperature. Specifically, the rate of O_3_ concentration increase reached 15.0 μg m^−3^ °C^−1^ between 26–28 °C and 30–32 °C temperature ranges. This rate significantly decreased to 4.14 μg m^−3^ °C^−1^ across the 32–34 °C and 34–36 °C temperature ranges. In contrast, the concentrations of NO_x_ and anthropogenic NMHCs exhibited a decrease with rising temperature (Figure 3b,c), aligning with the diurnal variation patterns presented in Figure 2. This finding can be attributed to the combined effects of enhanced dilution resulting from a higher boundary layer and increased photochemical removal driven by strong solar radiation during midday and early afternoon. Furthermore, isoprene levels increased with rising temperature within the 22–24 °C and 30–32 °C temperature ranges (Figure 3d), which is expected as high temperatures and solar radiation typically enhance biogenic emission rates [42].

#### 3.1.3. Wind Analysis of O_3_ and Its Precursors

**Figure 4:**
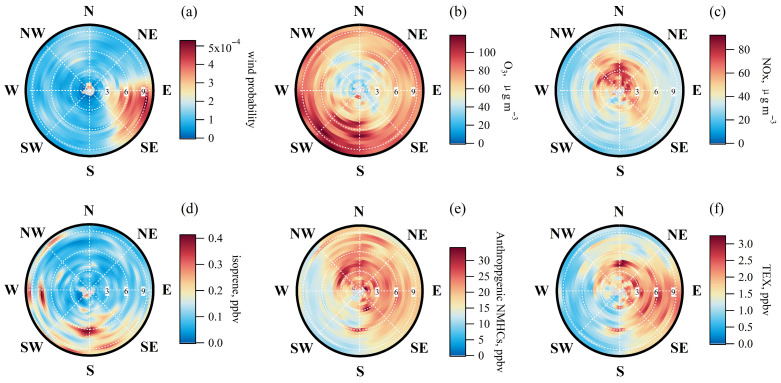
Influence of wind direction and speed on O_3_, NO_x_, isoprene, anthropogenic non-methane hydrocarbons (NMHCs), and TEX (the sum of toluene, ethylbenzene, and xylenes), as determined by Non-Parametric Wind Regression (NWR) analysis in ZeFir toolkit [43].

We investigated the influence of wind speed and directions on O_3_ and its precursors using the ZeFir toolkit [43]. The wind rose analysis for April–September in 2020 and 2021 revealed that the prevailing winds originated from the east and southeast (90–145°) (Figure 4a). O_3_ concentrations showed a distinct relationship with wind speed: lower levels were observed at speeds below 3 km h^−1^, while higher concentrations occurred at speeds exceeding 6 km h^−1^. Although elevated O_3_ was recorded from all directions, the highest values were associated with southerly winds (135–225°) (Figure 4b). In contrast, NO_x_ concentrations were inversely related to O_3_, peaking under low wind speeds (<6 km h^−1^) from the north (Figure 4c). This suggests that at lower wind speeds, locally emitted NO_x_ possibly accumulated and depleted O_3_ through titration. Isoprene and anthropogenic NMHCs also exhibited unique patterns based on wind conditions. High isoprene concentrations were predominantly observed with southerly winds exceeding 5 km h^−1^ (Figure 4d). Conversely, elevated anthropogenic NMHCs were recorded from a broader range of wind directions, from northwest to south (0–180° and 315–360°), under both low and high wind speeds (Figure 4e), indicating a potential influence from air mass transport. This pattern was further clarified by specific tracers for solvents and paint use—toluene, ethylbenzene, and xylenes [44]. At low wind speeds, their summed concentrations (TEX) were highest from the northwest-to-south sector (Figure 4f). Crucially, under high wind speeds, elevated levels of these compounds were also observed from the east and southeast, suggesting that their presence was possibly augmented by regional transport from these specific directions.

### 3.2. Air Mass Transport Pathways in Urban Nanjing

**Figure 5:**
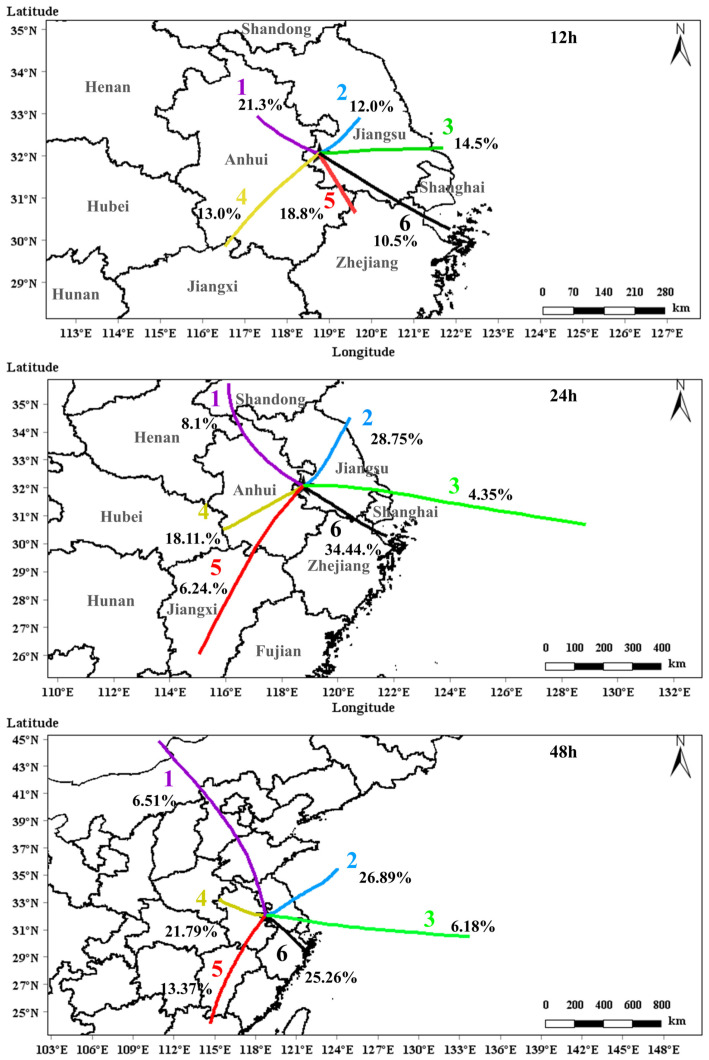
Six clusters of 12 h, 24 h, and 48 h air mass backward trajectories and their relative contributions. Trajectories were obtained from MeteoInfo for urban Nanjing during April–September of 2020 and 2021. The colored lines indicate clusters of air mass trajectories.

Figure 5 illustrates the six clusters of 12 h, 24 h, and 48 h backward-trajectory affecting urban Nanjing during April–September of 2020 and 2021 obtained using the MeteoInfo. The six clusters of 12 h backward trajectories indicate that all air mass origins were located within the YRD region. The Cluster 1 originated from the northwest of Nanjing, traveled a short distance across northern Anhui Province to reach the NJ site, and accounted for 21.3% of total air mass trajectories. The Cluster 2, representing air masses from the northeast that traversed central Jiangsu Province, constituted 12.0% of total trajectories. Originating east of Nanjing, the Cluster 3 tracked back to the southeast coastal area and traversed the southern Jiangsu Province, representing 14.5% of the total trajectories. The Cluster 4 originated from the southwest, with its source region in southern Anhui Province, and accounted for 13.0% of the total trajectories. The Clusters 5 and 6 both originated from the southeast of the YRD regions but exhibited different transport distances and source locations. The Cluster 5 followed a more southerly and shorter transport path, originating near the intersection of Jiangsu, Zhejiang, and Anhui provinces, and accounted for 18.8% of air mass trajectories. In contrast, the Cluster 6 involved long-range transport from the East China Sea off the coasts of northern Zhejiang Province and Shanghai, passing through southern Jiangsu and northern Zhejiang Province before reaching NJ, accounting for 10.5% of all trajectories. Air masses from eastern and southeastern pathways (Clusters 3, 5, and 6) accounted for 43.8% of the total trajectories, consistent with the observed prevailing east and southeast winds during April–September based on ground wind speed and direction measurements (Figure 4).

Analysis of the 24 h backward trajectories indicated that, like the 12 h trajectories, the dominant air mass origin was the southeast (Cluster 6, 34.4%). However, a key distinction in the 24 h results was the substantial increase in northeasterly trajectories (Cluster 2, 28.8%), while contributions from the northwest (Cluster 1, 8.1%) and east (4.35%) decreased. Two separate southwesterly clusters were identified: a shorter-range trajectory from southern Anhui Province (Cluster 4, 18.1%) and a long-range trajectory from Jiangxi Province (Cluster 5, 6.24%). When extending the trajectory duration to 48 h, the contribution of long-range southwesterly transport (Cluster 5) more than doubled to 13.4%. Furthermore, the short-range southwesterly air mass observed in the 24 h analysis (Cluster 4) shifted, becoming a short-range northwesterly-by-westerly cluster that accounted for 21.8% of the 48 h backward trajectories.

In this study, we focused on the influence of air mass transport on daytime (10:00–16:00) O_3_ concentrations. During this period, the reaction with OH radical is the dominant removal pathway for NMHCs [45]. Assuming the OH-oxidation of NMHCs follows a pseudo-first-order process, the atmospheric lifetimes (τ) of NMHCs can be calculated using the following equation:

(5) $$\tau_{i}=\frac{1}{\left[OH\right]k_{OH,i}}$$

where [OH] represents the average concentration of OH radical during 10:00–16:00, which was assumed to 5 × 10^6^ molecule cm^−3^ according to the OBM-simulated OH concentrations, which is consistent with the value for Shanghai [45]; k_OH,i_ denotes the reaction rate constant of species i with OH radical. Based on this assumption, the τ values for the 57 NMHCs ranged from 0.83 to 224 h. Notably, 47 of these species (approximately 80%) had τ values shorter than 12 h. Consequently, given the short atmospheric lifetime of many NMHCs, we selected the 12 h backward trajectory results for our analysis of air mass transport’s influence on O_3_ and its precursors.

### 3.3. Characteristics of O_3_ and Its Precursors During Four O_3_ Pollution Episodes

#### 3.3.1. Temporal Variations in Concentrations of O_3_ and Its Precursors

**Figure 6:**
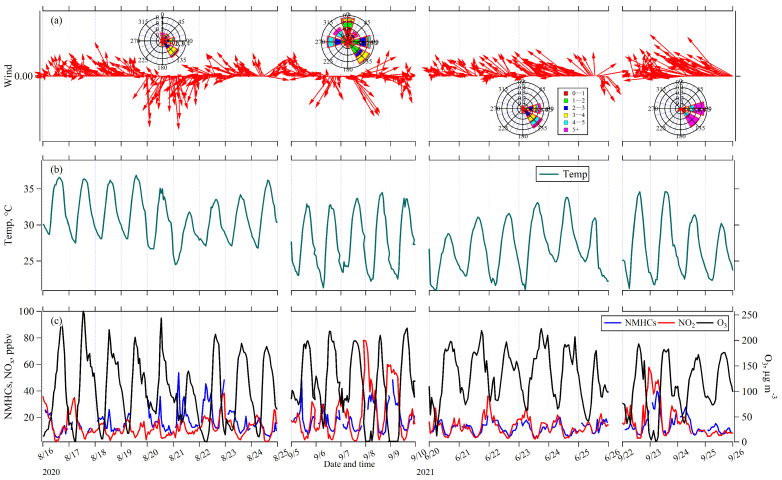
Temporal variations of (**a**) wind, (**b**) temperature, and (**c**) O_3_, NMHCs, and NO_2_ concentrations during the four O_3_ pollution episodes.

In this study, four O_3_ pollution episodes were selected for further analyses to evaluate the influence of air mass transport on O_3_ and its precursors NMHCs and NO_2_: 16–24 August 2020 (Episode I); 5–9 September 2020 (Episode II); 20–25 June 2021 (Episode III); and 22–26 September 2021 (Episode IV). Figure 6 presents the temporal variations of wind, temperature, O_3_, NMHCs, and NO_2_ concentrations during these four episodes. During four episodes, O_3_ exhibited a diurnal pattern characterized by elevated concentrations during early afternoon hours (13:00–15:00) and lower concentrations during nighttime (01:00–06:00). These patterns are consistent with typical photochemical O_3_ production driven by solar radiation [46]. In contrast, NO_2_ and NMHCs peaked during nighttime and early morning, showing an inverse diurnal variation pattern compared to O_3_. Hourly O_3_ concentrations exceeding 160 μg m^−3^ were recorded during these four episodes, with exceeding hours of 55 h, 37 h, 53 h, and 27 h, respectively. The average O_3_ concentrations during these hours for Episodes I through IV were 187, 194, 184, and 174 μg m^−3^, respectively. Meanwhile, the highest average NMHCs mixing ratio (12.80 ppbv) was observed in Episode I, followed by Episode II (11.40 ppbv), Episode III (8.95 ppbv), and Episode IV (8.92 ppbv). Conversely, the average NO_2_ mixing ratios peaked in Episode III (9.5 ppbv), followed by Episode IV (7.7 ppbv), Episode I (7.2 ppbv), and Episode II (6.2 ppbv). These results indicated that O_3_ concentrations were not linearly related to NMHCs and NO_2_, highlighting the intricate influences from air mass transport or O_3_ formation sensitivity.

**Figure 7:**
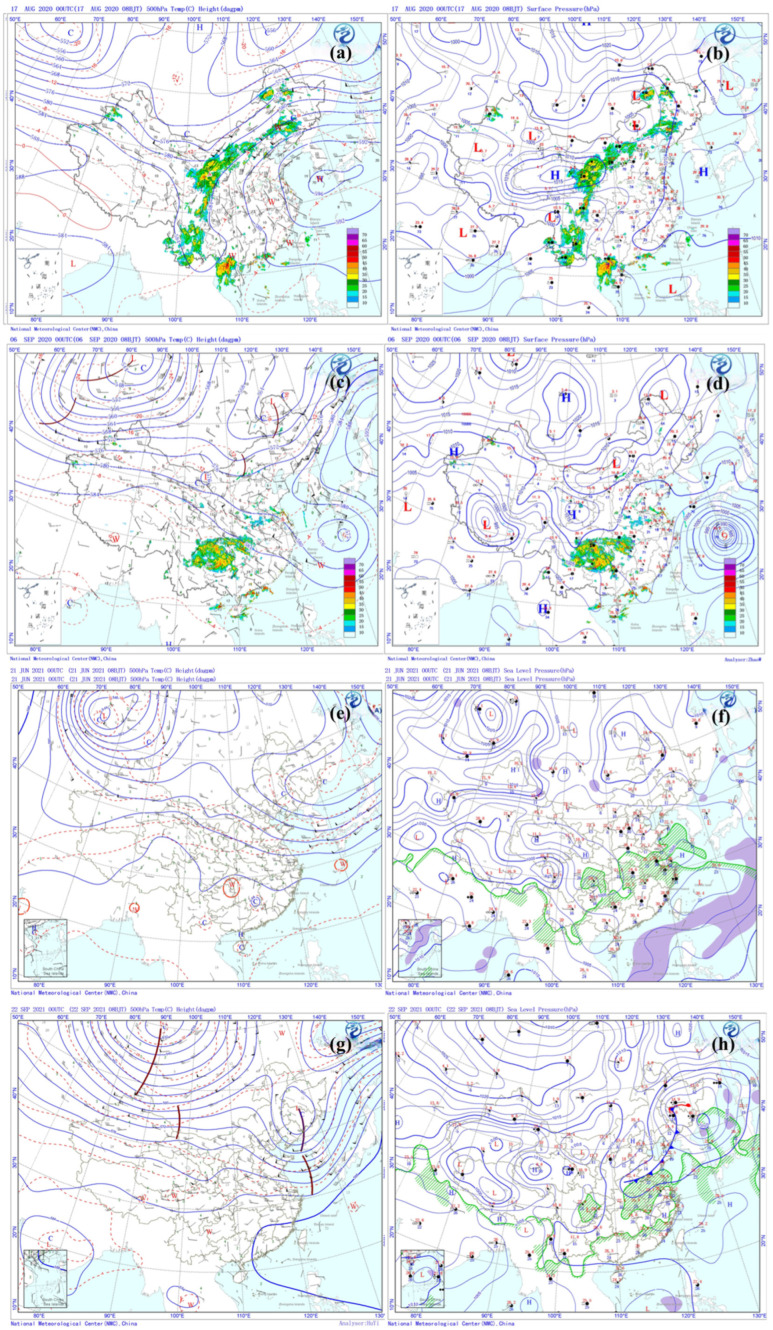
The 500 hPa and corresponding surface weather charts for (**a**,**b**) 17 August 2020, (**c**,**d**) 5 September 2020, (**e**,**f**) 21 June 2021, and (**g**,**h**) 22 September 2021. These charts were acquired from the China Meteorological Administration (https://www.wmc-bj.net (accessed on 29 June 2025)).

As depicted in Figure 7a, Nanjing and the YRD region were under the influence of a strong western Pacific subtropical high at 500 hPa on 17 August 2020, during Episode I. This dominant high-pressure system created conditions highly conducive to O_3_ formation, including strong sinking air, clear skies, and weak vertical mixing. Concurrently, the surface weather chart (Figure 7b) indicates that Nanjing was situated at the rear of a weak high-pressure system, experiencing prevailing southeasterly winds. These winds played a crucial role in transporting O_3_ and its precursors from the southeastern YRD towards Nanjing, aligning with the observed wind directions at monitoring sites (Figure 6a).

For 5 September 2020, during Episode II, Nanjing was located on the northwestern edge of the outer circulation of Super Typhoon Haishen at 500 hPa (Figure 7c). This positioning resulted in the area being affected by northwesterly winds and significant downward air movement, which effectively limited vertical mixing. At the surface, Figure 7d shows conditions dominated by a uniform pressure field with light southeast and southwest winds. These stable surface conditions were unfavorable for the horizontal dispersion of O_3_ and its precursors near the ground. During this episode, the average wind speed (2.33 m/s) was the lowest among the four episodes, and wind direction was complex, with influences from the north, southeast, and east (Figure 6a).

Episodes III (21 June 2021) and IV (22 September 2021) exhibited similar 500 hPa weather patterns (Figure 7e,g). Nanjing and the YRD region were influenced by northwesterly flow, situated behind a moving upper trough. This atmospheric configuration led to strong sinking motion and a prominent temperature inversion, both of which severely restricted the vertical dispersion of air pollutants. The accompanying descending dry air further enhanced clear-sky radiation. At the surface, the weather charts (Figure 7f,h) simultaneously show distinct but consistently stable atmospheric conditions. On 21 June Nanjing experienced a weak saddle-shaped pressure field, while on 22 September, it was situated within a weak warm sector ahead of a front. The predominant winds during these two episodes were both from the southeast and the east (Figure 6a).

#### 3.3.2. Sensitivity of O_3_ Formation to NMHCs and NO_x_

**Figure 8:**
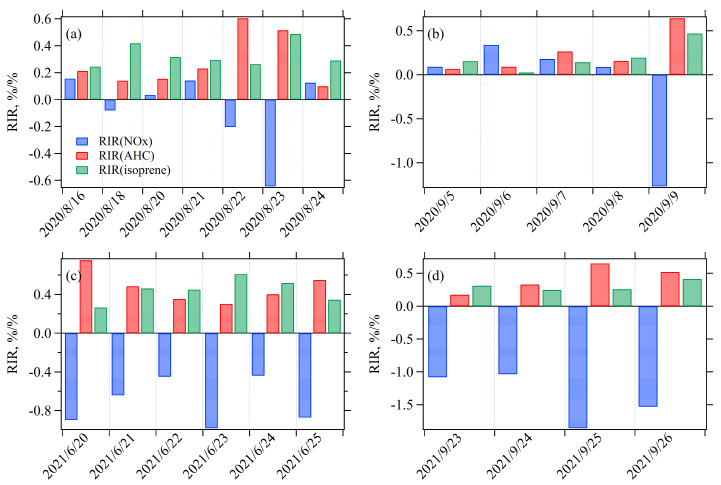
Daily average relative incremental reactivity (RIR) values of NO_x_, anthropogenic NMHCs (AHC), and isoprene during the four O_3_ pollution episodes.

Figure 8a–d displays the average RIR values of NO_x_, isoprene (mainly from biogenic emissions), and anthropogenic hydrocarbons (AHC) during four O_3_ episodes. During Episode I, the RIR(NO_x_) was negative for three days (18, 22, and 23 August 2020), and the RIR(NO_x_)/RIR(AHC) was 0.23 on August 20, 2020, indicating NMHCs-limited O_3_ sensitivity on these four days. For the other three days, the RIR(NO_x_)/RIR(AHC) ratios ranged from 0.62 to 1.27, suggesting the O_3_ sensitivity in the transition regime. During Episode II, the RIR(NO_x_) was negative on 9 September 2020 (indicating NMHCs-limited regime), while the RIR(NO_x_)/RIR(AHC) ratios ranged from 0.56 to 1.36 on three days (transition regime) and reached 3.8 on September 6, 2020 (NO_x_-limited regime). During Episodes III and IV, RIR(NO_x_) was negative on all days, suggesting NMHCs-limited O_3_ sensitivity. Furthermore, across the four episodes, RIR(isoprene) approximated RIR(AHC) on 10 days (with ratios of 0.7–1.3), yet surpassed it on seven days (with ratios > 1.3). These findings highlight the significant contribution of biogenic emissions to O_3_ formation, with biogenic reactivity reaching comparable or exceeding levels of anthropogenic NMHCs during certain days. This suggests that under specific atmospheric conditions, biogenic emissions can play an equally, or even more, critical role than anthropogenic pollutants in driving O_3_ formation. This vital insight for O_3_ control strategies underscores the need to consider both anthropogenic and biogenic influences.

### 3.4. Potential Source Regions of O_3_ and NMHCs

**Figure 9:**
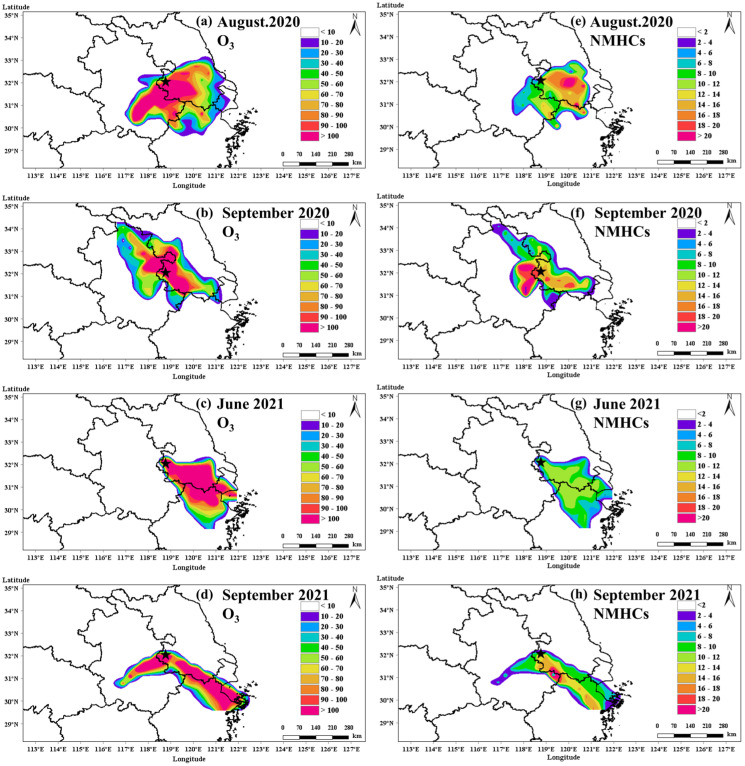
Spatial distribution of WCWT values for O_3_ (**a**–**d**) and NMHCs (**e**–**h**) during four O_3_ pollution episodes. The star symbol indicates the location of the observation site NJ.

Figure 9a–d presents the spatial distribution of WCWT values for O_3_ during the four O_3_ pollution episodes. During Episode I, high WCWT values for O_3_ were distributed across a broad area to the east, southeast, south, and southwest of the NJ site, extending from the southeastern coastal region of Jiangsu Province to southern Anhui Province (Figure 9a). In contrast, O_3_ WCWT hotspots during Episode II were primarily located northwest and southeast of the NJ site (Figure 9b). During Episodes III and IV, the high WCWT regions were primarily situated to the southeast, encompassing major urban areas along the Yangtze River in Shanghai, southern Jiangsu Province, and northern Zhejiang Province (Figure 9c,d). Compared to Episodes I and II, the contributions from northern regions were notably lower in Episodes III and IV. Additionally, Episode IV showed a secondary WCWT hotspot to the southwest of the NJ site.

The spatial distribution of WCWT values for NMHCs exhibited some differences from that of O_3_. In Episode I, the NMHCs WCWT hotspot was primarily concentrated to the east of the NJ site, with no high values were observed in the southwest despite elevated O_3_ contributions from that direction (Figure 9e). This discrepancy likely arises because southwestern areas like southern Nanjing and southern Anhui Province, characterized by high vegetation cover and limited anthropogenic activity [47], are unlikely major source regions of total NMHCs, which are influenced by both biogenic and anthropogenic sources [48]. Nonetheless, biogenic NMHCs such as isoprene, emitted during air mass transport across vegetated regions, can undergo photochemical reactions and enhance O_3_ formation. In Episode II, high NMHCs WCWT values were concentrated to the west, south, and southeast of the NJ site, particularly in urban and industrial areas such as Changzhou, Suzhou, and Wuxi in Jiangsu Province (Figure 9f). During Episodes III and IV, NMHCs hotspots were also observed to the southeast, encompassing southern Jiangsu Province, northern Zhejiang Province, and Shanghai (Figure 9g,h). Consistent with the emission inventory developed by An et al. (2021) [48], these regions are recognized as non-methane VOCs (NMVOCs) and NO_x_ emission hotspots along the Yangtze River, with significant contributions also from power plants and the automotive sector in central and eastern Anhui Province. These emisison inventory findings are largely consistent with the WCWT analyses in this study. However, it is also evident that variations in air mass back trajectories can lead to distinct shifts in the sources impacting the NJ site. Overall, the findings suggest that O_3_ and its precursor concentrations in urban Nanjing were strongly influenced by intra-regional transport within the Yangtze River Delta during these episodes, with industrial emissions from the southeast playing a particularly important role in elevating both O_3_ and NMHCs levels.

## 4. Discussion

### 4.1. Influence of Air Mass Transport on O_3_ and Its Precursors Levels

**Figure 10:**
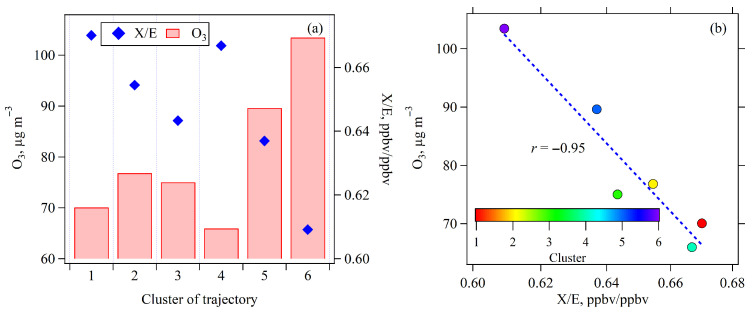
(**a**) Average O_3_ concentrations and o-xylene/ethylbenzene (X/E) ratios for six trajectory clusters during 10:00–16:00; (**b**) correlation between O_3_ concentrations and X/E ratios.

Figure 10a displays the average O_3_ concentrations observed between 10:00 and 16:00 during April–September of 2020 and 2021, categorized by the six identified air mass 12 h trajectory clusters. Notably, Cluster 6, originating from the east–southeast and characterized by long-distance back trajectories, exhibited the highest average O_3_ concentration (100 μg m^−3^), closely followed by Cluster 5 from the southeast (90 μg m^−3^). These results aligned with the high O_3_ WCWT values observed in the southeast regions across the four O_3_ pollution episodes (Figure 9). In contrast, Cluster 1, Cluster 2, and Cluster 3, originating from the north and northeast, showed moderate O_3_ levels (70–78 μg m^−3^), while Cluster 4 from the southwest recorded the lowest O_3_ concentration (65 μg m^−3^).

To further explore the relationship between air mass transport and O_3_ concentrations, the average ratios of o-xylene to ethylbenzene (X/E) during 10:00–16:00 were calculated for each trajectory cluster to indicate its photochemical aging degree. Considering that the emission ratio of o-xylene and ethylbenzene is relatively consistent, and given that o-xylene has a higher reactivity with OH radical than ethylbenzene, a lower X/E therefore indicates that air masses have undergone a longer period of atmospheric aging [49,50]. As presented in Figure 10b, average O_3_ concentrations exhibited a significant negative correlation with X/E ratios across the six trajectory clusters (r = −0.95, *p* < 0.05). This strong inverse relationship demonstrates that as air masses age (indicated by lower X/E values), O_3_ concentrations tend to increase. This pattern strongly suggests that photochemical reactions occurring during air mass transport are one of the important drivers of O_3_ formation.

**Figure 11:**
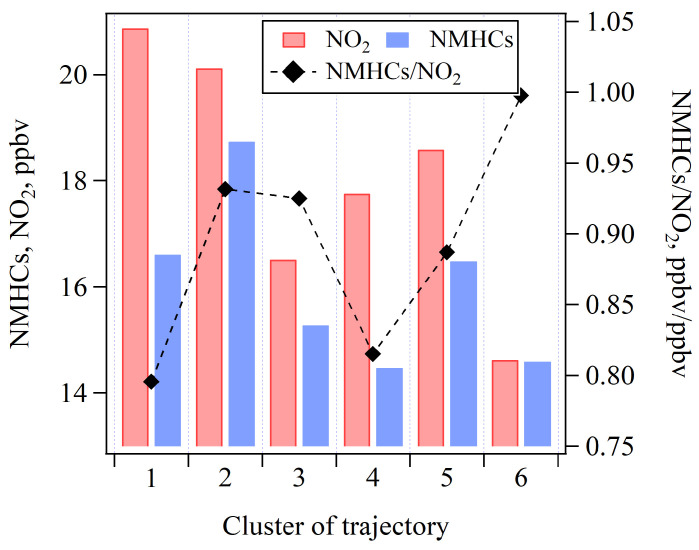
Average concentrations of NO_2_, NMHCs, and the NMHCs/NO_2_ ratio under six 12 h trajectory clusters during 10:00–16:00.

Figure 11 illustrates the average concentrations of NO_2_ and NMHCs, as well as the average ratio of NMHCs/NO_2_, for the six 12 h trajectory clusters. A comparison of Figure 10a and Figure 11 reveals that O_3_ levels are not directly determined by the absolute concentrations of its precursors. Notably, Cluster 6, which was associated with the highest O_3_ concentrations, exhibited lower levels of NO_2_ (14 ppbv) and NMHCs (14 ppbv) compared to the other trajectory clusters. However, this trajectory cluster exhibited the highest ratio of NMHCs/NO_2_. This may be attributed to the long transport distance of air mass, leading to differential photochemical aging of NMHCs and NO_2_, and potentially the spatial distribution of emission sources. Previous studies have shown that higher ratios of NMHCs/NO_2_ promote more efficient O_3_ formation due to enhanced radical propagation chains [4,51]. Consequently, the elevated NMHCs/NO_2_ ratio in Cluster 6 likely enhances O_3_ formation. In contrast, Clusters 1, 2, and 5 exhibited the highest NO_2_ and NMHCs concentrations. These air masses were associated with the shortest air mass transport distances (Figure 5), suggesting more stagnant meteorological conditions that potentially hinder air pollutants dispersion and lead to the accumulation of locally emitted NMHCs and NO_x_. However, despite higher precursor concentrations, the lower aging extents of air masses (Figure 10b) imply limited photochemical processing, thereby resulting in lower O_3_ levels in these three trajectory clusters. An exception was Cluster 5, which exhibited high concentrations for both O_3_ and its precursors, underscoring the significance of local photochemical O_3_ formation. Conversely, Clusters 1 and 4 showed the lowest NMHCs/NO_2_ ratios, aging extents of air mass, and O_3_ concentrations, thereby further highlighting the critical role of photochemical reactions during air mass transport in determining O_3_ levels.

### 4.2. Influence of Air Mass Transport on O_3_ Sensitivity to Its Precursors

#### 4.2.1. Comparison of O_3_ Sensitivity Across Six Trajectory Clusters

**Figure 12:**
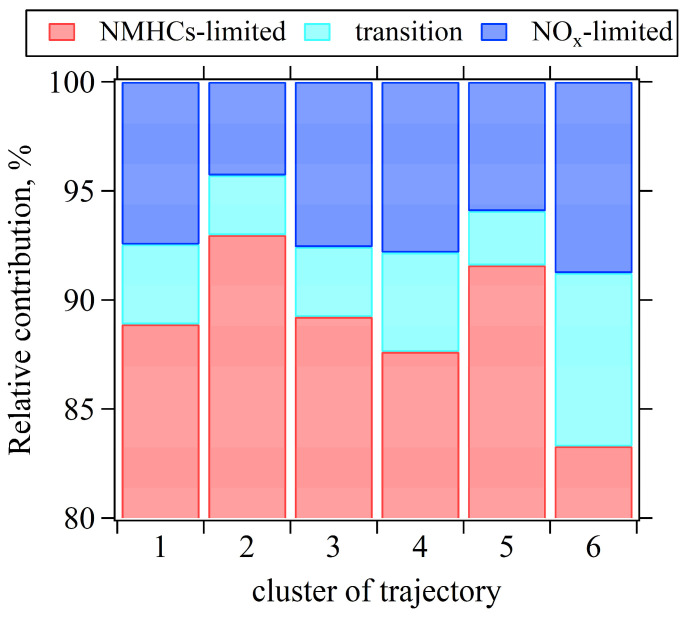
Relative contribution of NO_x_-limited, NMHCs-limited, and transition O_3_ sensitivity regimes under six 12 h trajectory clusters.

Simulations of O_3_ formation were conducted using the OBM for the daytime hours of a total of 312 days (146 days in 2020 and 162 days in 2021). The RIR values of NO_x_ and AHC were calculated for each hour between 08:00 and 18:00. Subsequently, O_3_ sensitivity in each hour was classified into three types (NMHCs-limited, NO_x_-limited, and transition regime) based on the classification criteria recommended by Xu et al. (2022) [40]. Figure 12 presents the relative contributions of three O_3_ sensitivity regimes across the six trajectory clusters, revealing that O_3_ sensitivity during daytime hours was dominated by the NMHCs-limited regime, accounting for 83% to 93% of the time for each trajectory cluster. Nevertheless, Cluster 6, originating from the southeast, showed the highest frequency of the transition (9%) and NO_x_-limited (8%) O_3_ sensitivity regimes. This is likely because this air mass trajectory crossed major NMHCs source regions and had a longer transport distance, resulting in a relatively higher NMHCs/NO_2_ ratio due to faster degradation of NO_x_. Similarly, Cluster 4 also showed a relatively higher frequency of transition and NO_x_-limited O_3_ sensitivity regimes (12%), likely influenced by the passage of air masses over forested regions (with abundant biogenic NMHCs emissions) and long transport distances. In contrast, Clusters 2 and 5 showed the lowest occurrence of transition and NO_x_-limited O_3_ sensitivity regimes (7–8%). This likely attributes to their short transport distances and strong influence from urban vehicular emissions.

#### 4.2.2. Air Mass Transport Influence on O_3_ Sensitivity During Four O_3_ Pollution Episodes

**Figure 13:**
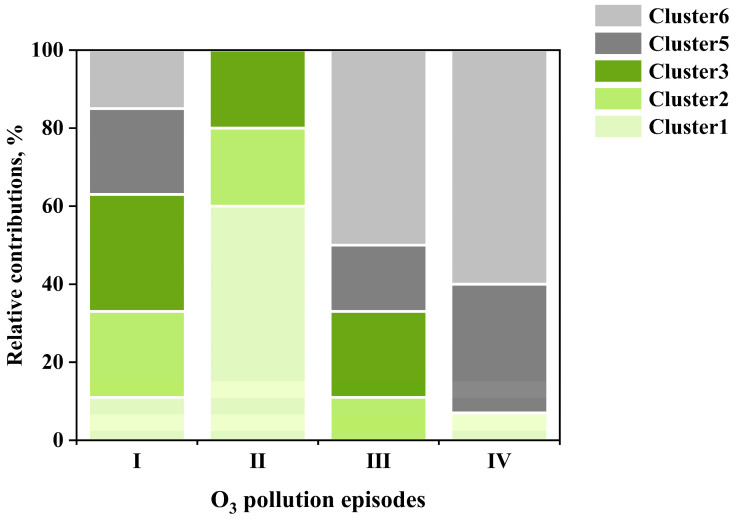
Relative contribution of six 12 h trajectory clusters during the O_3_ peak period (13:00–15:00) across the four episodes.

Figure 13 compares the relative contributions of the six backward 12 h trajectory clusters during the O_3_ peak periods (13:00–15:00) across the four O_3_ pollution episodes (Section 3.3). While Cluster 4, originating from the southwest, had negligible contributions during all O_3_ pollution episodes, the other five trajectory clusters exhibited variable contributions across different episodes. In Episode I, five trajectory clusters were involved, with origins predominantly from the east and north (Clusters 1–3, 63%), and the remaining from the southeast (Clusters 5 and 6, 37%). Episode II was dominated by transport from the northwest (Cluster 1; 60%), followed by contributions from the northeast (Cluster 2; 20%) and east (Cluster 3; 20%). By contrast, Episodes III and IV during their peak O_3_ periods were primarily influenced by southeastern transport pathways. Specifically, Clusters 5 and 6 together accounted for 67% of air masses in Episode III and a remarkable 93% in Episode IV. Minor contributions in Episode III came from easterly (Cluster 3, 22%) and northeasterly (Cluster 2, 11%) transport, while Episode IV included a small fraction (7%) from the northwest (Cluster 1). These differing air mass origins during these episodes corresponded to distinct O_3_ sensitivity regimes. As discussed earlier (Figure 8), Episodes I and II displayed mixed O_3_ sensitivity regimes—some days they exhibited NMHCs-limited O_3_ sensitivity, while others fell into transition or NO_x_-limited regimes. This variability may be attributed to the complexity of air mass origins during these episodes (Figure 13). Conversely, Episodes III and IV, dominated by southeast air mass transport (Clusters 5 and 6), consistently exhibited NMHCs-limited O_3_ sensitivity. This disparity in sensitivity regimes further confirms the significant influence of air mass transport in shaping the sensitivity of O_3_ formation to its precursors.

### 4.3. Limitations and Uncertainties

In this study, we employed the WCWT and OBM approaches to investigate the transport of O_3_ and its precursors, as well as the sensitivity of O_3_ formation. However, several sources of uncertainty may affect the WCWT results. These include potential errors in trajectory calculations due to the resolution of meteorological input data and inherent assumptions in the trajectory model. For example, the assumption that pollutant concentrations are solely transported along back trajectories may overlook atmospheric chemical transformations and removal processes, particularly for reactive species such as NMHCs and NO_x_. To mitigate these uncertainties, we tested multiple trajectory lengths (12, 24, and 48 h; Figure 5) and cross-validated the WCWT results with known regional emission characteristics reported by An et al. (2021) [48] and prevailing wind directions (Figure 6), thereby enhancing the robustness of the identified potential source regions.

In addition to WCWT and OBM, air quality models based on emissions, such as CMAQ and WRF-Chem, have been widely applied to study O_3_ formation and transport in the YRD region. For instance, Wang et al. (2023) found that both NO_x_ and NMHCs had strong transport potential, and cities including Nanjing were primarily under NMHCs-limited O_3_ formation regimes [52]. Li et al. (2021) demonstrated that NMHCs sources contributed more significantly to O_3_ formation than NO_x_ [53], further supporting the NMHCs-limited regime identified by our OBM results. Hu et al. (2018) reported that regional transport substantially contributed to O_3_ pollution, even under unfavorable local meteorological conditions [54]. Furthermore, Li et al. (2021) employed lidar measurements to observe O_3_ transport from Shanghai to other areas within the YRD region, resulting in elevated regional O_3_ levels [21]. These findings support our WCWT-based conclusion that regional transport of O_3_ and its precursors plays a critical role in O_3_ pollution within the YRD region.

We also used the RIR of isoprene calculated using the OBM to assess the impact of biogenic emissions on O_3_ formation. However, this method only provides a preliminary estimate, and a more comprehensive assessment of biogenic NMHCs, including monoterpenes and other species, and their interactions with anthropogenic emissions is needed to fully characterize their role in O_3_ formation. Future studies incorporating receptor modeling (e.g., positive matrix factorization (PMF) or chemical mass balance (CMB) models), high-resolution emission inventories, and chemical transport modeling would significantly improve the accuracy of source apportionment for O_3_ and its precursors. Given the complexity and spatiotemporal variability of NMHC emissions, such investigations would be a valuable direction for future research.

## 5. Conclusions

During April–September in 2020 and 2021, the average concentrations for DMA-1h O_3_, NMHCs, and NO_x_ were 149 ± 50.5 μg m^−3^, 16.7 ± 10.7 ppbv, and 31.7 ± 25.5 μg m^−3^, respectively. The diurnal variation analyses indicated that O_3_ and isoprene concentrations peaked during the midday and early afternoon, while NO_x_ and anthropogenic NMHCs presented their lowest observed values. Isoprene showed poor correlation with acetylene, suggesting its primary origin from biogenic emissions. O_3_ and isoprene concentrations displayed a positive, yet non-linear, relationship with temperature, whereas NO_x_ and NMHCs decreased when temperature rose. Wind analysis further indicated the potential influence of air mass transport on O_3_ and its precursors.

Cluster analyses of 12 h, 24 h, and 48 h backward-trajectories consistently identified the east and southeast as the dominant air mass transport pathway. Given our focus on air transport within the YRD region and eastern China, and considering the relative short lifetimes of NMHCs and NO_x_, the six clusters derived from 12 h backward trajectories were selected to discuss impact of air mass transport on O_3_ and its precursors. Two southeastern trajectories (Clusters 5 and 6) were the most dominant air mass transport pathways, together accounting for 29.3%, followed by eastern (26.5%), northwestern (21.3%), southwestern (13.0%), and northeastern (12.0%) pathways. Notably, the southeastern clusters were associated with the highest O_3_ concentrations (100 μg m^−3^ and 90 μg m^−3^, respectively), significantly surpassing values observed for other trajectory clusters (65–78 μg m^−3^). Furthermore, O_3_ concentrations exhibited a strong negative correlation with the X/E ratios, signifying that higher O_3_ levels were associated with more photochemically aged air masses. This finding suggested that O_3_ concentrations were not directly determined by the levels of NMHCs or NO_2_. Instead, long-range transport trajectory clusters were characterized by lower absolute O_3_ precursor concentrations but exhibited higher NMHCs/NO_2_ ratios and consequently higher O_3_ levels, whereas shorter-range trajectory clusters showed elevated precursor concentrations but lower O_3_ levels. This pattern underscores the critical role of photochemical reactions in air mass transport for O_3_ accumulation.

To evaluate the impact of air mass transport on O_3_ sensitivity, four O_3_ pollution episodes with peak concentrations exceeding 160 μg m^−3^ were selected. While the controlling synoptic systems varied—with Episode I dominated by a 500 hPa subtropical high and Episodes II–IV by post-low-pressure circulation—all four episodes shared critical meteorological characteristics. Specifically, each featured strong downward airflow and suppressed vertical diffusion, which are conditions highly favorable for the formation and accumulation of O_3_. The air mass origins differed between the O_3_ pollution episodes for these two years; the 2020 episodes involved a more diverse mix of southeastern and northern transport pathways, whereas the 2021 episodes were predominantly influenced by southeastern transport pathways. The WCWT analysis results confirmed these findings, showing that high O_3_ contributions originated from the southeast, south, and southwest during Episode I, and from the northwest and southeast during Episode II. For Episodes III and IV, high contributions were mainly associated with transport from urban-industrial regions in southeastern Jiangsu Province, northern Zhejiang Province, and Shanghai. Mapping potential NMHCs source regions revealed a partial spatial overlap with O_3_ concentrations. However, distinct features were also evident, particularly in vegetated areas, suggesting a significant role for biogenic emissions in O_3_ formation.

The OBM simulations further revealed that O_3_ formation across all six trajectories clusters were primarily in the NMHCs-limited regime (accounting for 83–93% of daytime hours). However, Cluster 6 (southeastern long-range transport) exhibited the highest frequency of the transition (9%) and NO_x_-limited (8%) regimes. This can be attributed to the relatively high NMHCs/NO_2_ ratios in these aged air masses and to the passage through regions featuring substantial anthropogenic NMHCs emissions. Cluster 4 also presented relatively higher frequency of transition and NO_x_-limited O_3_ sensitivity, likely due to biogenic emissions. In contrast, Clusters 2 and 5, characterized by shorter transport distances and a strong influence from urban vehicular emissions, demonstrated the lowest occurrence of these sensitivity regimes. Further supporting these findings, analysis of four selected O_3_ pollution episodes revealed that the two episodes in 2020, associated with more complex air mass trajectory combinations, exhibited varied O_3_ sensitivity regimes, while the 2021 episodes, dominated by southeastern air masses, were consistently NMHCs-limited. Collectively, these results corroborate that O_3_ sensitivity in Nanjing is significantly modulated by the origin and transport characteristics of incoming air masses. This study demonstrates that regional air mass transport plays a important role not only in determining O_3_ and precursor concentrations but also in shaping the sensitivity of O_3_ formation, highlighting the necessity of implementing regionally coordinated control strategies for effective mitigation of O_3_ pollution across the YRD in China.

## Data Availability

Researchers wishing to access the data used in this study can make a request to the corresponding author: wangming@nuist.edu.cn.

## Abbreviations

O_3_	ozone
SO_2_	sulfur dioxide
VOCs	volatile organic compounds
NO_x_	nitrogen oxides
NO	nitric oxide
NO_2_	nitrogen dioxide
CO	carbon monoxide
YRD	the Yangtze River Delta
PRD	the Pearl River Delta
CWT	concentration-weighted trajectory
OBM	observation-based box model
GC–MS	gas chromatography–mass spectrometry
GDAS	global data assimilation system
WCWT	weighted concentration-weighted trajectory
CB05	carbon bond lumped chemical reaction mechanism
RIR	relative incremental reactivity
NMHCs	non-methane hydrocarbons
AHC	anthropogenic non-methane hydrocarbons
X/E	ratio of o-xylene to ethylbenzene
NJ	Nanjing
DMA-1h	daily maximum 1 h moving average
DMA-8h	daily maximum 8 h moving average
TUV	tropospheric ultraviolet–visible radiation
IOA	index of agreement
TEX	the sum of toluene, ethylbenzene, and xylenes
NWR	non-parametric wind regression
RH	relative humidity

## Appendix A

**Table A1:** The measured 57 NMHC species in this study.

Alkanes		Alkenes and Alkynes	Aromatics
ethane	2,4-dimethylpentane	ethene	benzene
propane	2-methylhexane	propene	toluene
n-butane	3-methylhexane	1-butene	ethylbenzene
i-butane	cyclohexane	cis-2-butene	m,p-xylene
n-pentane	methylcyclopentane	trans-2-butene	o-xylene
i-pentane	n-octane	1,3-butidene	styrene
cyclopentane	2,2,4-trimethylpentane	1-pentene	i-propylbenzene
n-hexane	2,3,4-trimethylpentane	cis-2-pentene	n-propylbenzene
2,2-dimethylbutane	methylcyclohexane	trans-2-pentene	3-ethyltoluene
2,3-dimethylbutane	2-methylheptane	isoprene	4-ethyltoluene
2-methylpentane	3-methylheptane	1-hexene	2-ethyltoluene
3-methylpentane	n-nonane	acetylene	1,3,5-trimethylbenzene
n-heptane	n-decane		1,2,4-trimethylbenzene
2,3-dimethylpentane	n-undecane		1,2,3-trimethylbenzene
			1,3-diethylbenzene
			1,4-diethylbenzene
